# Thrombin, a mediator of cerebrovascular inflammation in AD and hypoxia

**DOI:** 10.3389/fnagi.2013.00019

**Published:** 2013-05-09

**Authors:** Debjani Tripathy, Alma Sanchez, Xiangling Yin, Jinhua Luo, Joseph Martinez, Paula Grammas

**Affiliations:** Garrison Institute on Aging, Department of Neurology, Texas Tech University Health Sciences CenterLubbock, TX, USA

**Keywords:** Alzheimer's disease, thrombin, hypoxia, neuroinflammation, endothelial cells, dabigatran

## Abstract

Considerable evidence implicates hypoxia and vascular inflammation in Alzheimer's disease (AD). Thrombin, a multifunctional inflammatory mediator, is demonstrable in the brains of AD patients both in the vessel walls and senile plaques. Hypoxia-inducible factor 1α (HIF-1α), a key regulator of the cellular response to hypoxia, is also upregulated in the vasculature of human AD brains. The objective of this study is to investigate inflammatory protein expression in the cerebrovasculature of transgenic AD mice and to explore the role of thrombin as a mediator of cerebrovascular inflammation and oxidative stress in AD and in hypoxia-induced changes in brain endothelial cells. Immunofluorescent analysis of the cerebrovasculature in AD mice demonstrates significant (*p* < 0.01–0.001) increases in thrombin, HIF-1α, interleukin-6 (IL-6), monocyte chemoattractant protein-1 (MCP-1), matrix metalloproteinases (MMPs), and reactive oxygen species (ROS) compared to controls. Administration of the thrombin inhibitor dabigatran (100 mg/kg) to AD mice for 34 weeks significantly decreases expression of inflammatory proteins and ROS. Exposure of cultured brain endothelial cells to hypoxia for 6 h causes an upregulation of thrombin, HIF-1α, MCP-1, IL-6, and MMP2 and ROS. Treatment of endothelial cells with the dabigatran (1 nM) reduces ROS generation and inflammatory protein expression (*p* < 0.01–0.001). The data demonstrate that inhibition of thrombin in culture blocks the increase in inflammatory protein expression and ROS generation evoked by hypoxia. Also, administration of dabigatran to transgenic AD mice diminishes ROS levels in brain and reduces cerebrovascular expression of inflammatory proteins. Taken together, these results suggest that inhibiting thrombin generation could have therapeutic value in AD and other disorders where hypoxia, inflammation, and oxidative stress are involved.

## Introduction

Alzheimer's disease (AD) is a complex, multifactorial neurodegenerative disease that affects more than 5.3 million people in the United States (Ballard et al., 2011; www.alz.org). The processes and factors that drive disease pathogenesis are not well delineated, although considerable literature suggests that vascular risk factors and vascular pathology are important (Farkas and Luiten, 2001; de la Torre, 2002; Knopman and Roberts, 2010; Grammas, 2011). Neurovascular abnormalities have been extensively documented in AD (Bell and Zlokovic, 2009) and abnormal vascular function could impact neuronal viability in multiple ways (de la Torre, 2000; Grammas, 2011; Sengillo et al., 2012). Inflammation and hypoxia are important mechanisms implicated in the development of AD (Kalaria, 2000; Grammas and Ovase, 2001, 2002; Peers et al., 2007; Grammas et al., 2011; Broussard et al., 2012; Carnevale et al., 2012).

In the brains of AD patients, as well as in animal models of AD, numerous reports document upregulation of a wide variety of inflammatory proteins (Rubio-Perez and Morillas-Ruiz, 2012; Zhang et al., 2013). Expression of proinflammatory cytokines and other inflammatory mediators are clearly demonstrable in regions of the brain most associated with AD pathology (Xia and Hyman, 1999). Also, a role for inflammation as a driver of pathologic events in the AD brain is supported by studies that show pharmacologic inhibition of inflammation through prolonged use of non-steroidal anti-inflammatory drugs significantly reduces the risk of developing AD (In't Veid et al., 2002; Etminan et al., 2003; Hoozemans et al., 2003). The cerebrovasculature is likely an active participant in neuroinflammation as a large number of inflammatory proteins including thrombin, tumor necrosis factor (TNFα ), interleukin (IL)-1, IL-6, IL-8, monocyte chemoattractant protein (MCP)-1, and matrix metalloproteinases (MMPs) are over expressed in AD-derived vessels compared to vessels from age-matched controls (Grammas and Ovase, 2001; Grammas et al., 2006, 2011; Thirumangalakudi et al., 2006).

Inflammation and hypoxia are interrelated processes; hypoxia can elicit tissue inflammation and inflamed tissues frequently become hypoxic (Himadri et al., 2010; Imtiyaz and Simon, 2010; Eltzschig and Carmeliet, 2011; Koeppen et al., 2011). Cerebral hypoperfusion, leading to hypoxia, has been implicated as an important underlying factor that promotes dementia (Peers et al., 2009; Daulatzai, 2012). Hypoxia triggers a cascade of events that contribute to the pathologic processes of AD through activation of multiple pathways (Sharp and Bernaudin, 2004; Zhang and Le, 2010). In this regard, hypoxia has profound effects on vasculature and has been shown to modulate endothelial reactive oxygen species (ROS) generation and to stimulate a pro-inflammatory gene expression (Giordano, 2005; Sanchez et al., 2012). The transcriptional factor hypoxia inducible factor (HIF)-1α, a primary sensor of low oxygen tension and a regulatory molecule which controls the cellular response to hypoxia, is elevated in the cerebromicrocirculation of AD patients and AD transgenic mice (Grammas et al., 2006, 2011). Finally, a link between cerebrovascular inflammation and hypoxia is further suggested by data showing that the inflammatory proteins elevated in the brain microvasculature of AD patients and/or AD mice are also increased in brain endothelial cells exposed to hypoxia (Grammas et al., 2011; Sanchez et al., 2013).

Many of the inflammatory proteins over expressed in the AD cerebrovasculature have detrimental effects on neurons. Thrombin, a multifunctional inflammatory mediator demonstrable in the brains of AD patients both in the vessel walls and senile plaques (Akiyama et al., 1992; Yin et al., 2010), is likely a central mediator of neuronal injury. Thrombin is directly toxic to neurons and can also potentiate neuronal injury indirectly via activation of neighboring microglia and astrocytes (Choi et al., 2003, 2005, 2008). In this regard, thrombin's neurotoxic properties have been extensively documented both *in vitro* and *in vivo* (Turgeon et al., 1999; Reimann-Philipp et al., 2001; Mhatre et al., 2004). Administration of thrombin directly into the rat brain results in neuronal cell death, glial scarring, and cognitive deficits (Mhatre et al., 2004). Also, exposure of microglia or astrocytes to thrombin results in increased release of noxious ROS and MMPs (Choi et al., 2005, 2008). Because thrombin, a key meditator of angiogenesis, is elevated in response to hypoxia (Landau et al., 2000), this protein could be an important regulator of the cerebrovascular response to hypoxia in AD.

The objective of this study is to investigate thrombin, HIF-1α, and inflammatory protein expression in the cerebrovasculature of transgenic AD mice and to explore the role of thrombin as a mediator of cerebrovascular inflammation in AD and hypoxia- mediated inflammation.

## Materials and methods

### Culture and hypoxic exposure of rat brain endothelial cells

Brain endothelial cell cultures were obtained from rat brain microvessels, as previously described (Diglio et al., 1993). The purity of these cultures was confirmed using antibodies to the endothelial cell surface antigen Factor VIII. Endothelial cells used in this study (passages 8–15) were maintained in Dulbecco's modified Eagle's medium (DMEM, Sigma-Aldrich, St. Louis, MO) supplemented with 10% fetal bovine serum (FBS), 1% antibiotic/antimycotic, and 2 mM glutamine. Confluent endothelial cell cultures were washed three times with Hank's balanced salt solution (HBSS, Gibco, Grand Island, NY) and then incubated at 37°C with serum-free DMEM for 6 h under hypoxic (1% O_2_) or normoxic (21% O_2_) conditions.

### Measurement of cell survival by MTT assay

Cells were washed with phosphate buffer saline (PBS) and incubated with the MTT reagent 3-(4,5-dimethylthiazol-2-yl)-2-5-diphenyl tetrazolium bromide (1:40 dilution) for 5–10 min at 37°C. The cells convert the MTT reagent to formazan which is quantified by colorimetric assay (Cell Titer 95 Aqueous solution cell proliferation assay, Promega, Madison, WI). The formazan product was read at 490 nm. The number of control cells, i.e., viable cells not exposed to any treatment, was defined as 100%.

### Administration of thrombin inhibitor to mice and immunofluorescent staining of mice brain sections

Adult wild-type 3xTgAD—LaFerla (control) and 3xTgAD—LaFerla mice were purchased at 8 weeks of age from The Jackson Laboratory (Bar Harbor, ME). Daily administration of the orally available direct thrombin inhibitor (DTI) dabigatran etexylate (Pradaxa®, Boehringer Ingelheim, Germany) (100 mg/kg) in PBS to AD mice and vehicle to control mice began at 18 weeks of age and continued daily for 34 weeks. DTI was administered in food. To ensure all food, and therefore drug, was consumed while maintaining *ad libitum* weight, mice were weighed and food intake monitored daily. All animal procedures were performed in accordance with NIH “Guide for the Care and Use of Laboratory Animals” and Texas Tech University Health Sciences Center Institutional Animal Care and Use Committee (IACUC) guidelines.

Mice were euthanized and brain tissue fixed with 10% neutral buffered formalin (NBF). The brain was removed and 1 mm blocks of tissue from cerebral cortex were post fixed in 10% NBF for additional 12 h and embedded in paraffin. Brain sections from the frontal cortex (7 μm thick) were deparaffinized in xylene, hydrated through a graded alcohol series, and then rinsed for 5 min in deionized water. Sections were subjected to heat-induced epitope antigen retrieval, washed with Tris-buffered saline with Tween (TBST) and blocked with 10% donkey serum at room temperature for 2 h. The sections were incubated at 4°C overnight with primary antibodies against HIF-1α (ab1, Abcam, Cambridge, MA), thrombin (sc16972, Santa Cruz Biotechnology, Santa Cruz, CA), MMP2 (ab37150, Abcam), IL-6 (ab6672, Abcam), MCP-1 (ab9858, Abcam), or the endothelial cell marker von Willebrand Factor (vWF, sc114014, sc8068, Santa Cruz) in TBS containing 2.5% donkey serum. Sections were then washed, blocked, and incubated with appropriate secondary antibodies conjugated with Alexa Fluor 488 or Alexa Fluor 594. Sections (3 per mouse) were incubated with DAPI solution at room temperature for 25 min and viewed using an Olympus IX71 microscope and quantified with HAMAMATSU imaging software.

### Detection of reactive oxygen species in cell culture and mouse brain tissue sections

Brain endothelial cell cultures were grown on coverslips in 24-well plates, incubated at 37°C in serum free media with or without dabigatran (DTI, 1 nM) and exposed to hypoxic (6 h, 1% O_2_) or normoxic (21% O_2_) conditions. Sections from frozen brain (10–20 μm thickness) were placed on coverslips in 24-well plates with 0.5 ml of PBS at room temperature. Brain sections and cell cultures were incubated with 5 μM of dihydroethidium (DHE) fluorescence dye (Life Technologies, Grand Island, NY; D23107) and Hoechst33342 NucBlue stain (Life Technologies, R37605) for 30 min in the dark at room temperature. DHE is a cell permeable compound which reacts with intracellular ROS to form oxyethidium, which emits a bright red color detectable by fluorescent microscopy. Following two washes with PBS, images were captured immediately using an Olympus IX71 microscope and analyzed using HAMAMATSU imaging software.

### Real-time PCR analysis

RNA from cultured rat brain endothelial cells was prepared using the TRI Reagent RT (Molecular Research Center, Inc., Cincinnati, OH), according to manufacturer protocol. Four micrograms of total RNA were reverse transcribed using oligo dT primers and Transcriptor high fidelity reverse transcriptase according to the manufactures protocol (Roche Applied Science). Real-time PCR was performed using the Applied Biosystems 7900 HT fast real time PCR system. Taqman gene expression master mix and Taqman gene expression assays from Applied Biosystems (Weiterstadt, Germany) were used for PCR. Primers for Thrombin (rat: Assay ID Rn00575908_m1, mice: Mm00438843_m1), HIF-1α (rat: Assay ID Rn00577560_m1, mice: Mm00468869_m1), MMP2 (rat: Assay ID Rn01538170_m1, mice: Mm00439498_m1), IL-6 (rat: Assay ID Rn01410330_m1, mice: Mm00446190_m1), MCP-1 (rat: Assay ID Rn00580555_m1, mice: Mm00441242_m1), and actin (rat: Assay ID Rn00667869_m1, mice: Mm00607939_s1) were used for these experiments. Results were normalized to actin. Fold difference between two samples (relative quantification) was determined by use of the delta–delta method [S1/S2 = 2 −(T1 − T2)], where S1 and S2 represent samples 1 and 2, and T1 and T2 represent the threshold cycles of samples 1 and 2, respectively.

### Statistical analysis

Data from each experiment are expressed as mean ± standard deviation (*SD*). The One-Way ANOVA followed by Bonferroni's comparison tests were performed for multiple samples. Statistical significance was determined at *p* < 0.05.

## Results

### Thrombin inhibition affects the inflammatory response of brain endothelial cells exposed to hypoxia

Brain microvessel endothelial cell cultures were incubated with the DTI dabigatran (10 pM–100 nM), exposed to hypoxia for 6 h and cell viability measured by MTT assay. There was a modest (19%) but significant (*p* < 0.05) increase in cell survival at 1–10 nM DTI while DTI doses in excess of 100 nM were toxic. Exposure of endothelial cultures to DTI under normoxic conditions did not affect cell survival at doses under 100 nM (data not shown).

**Figure 1:**
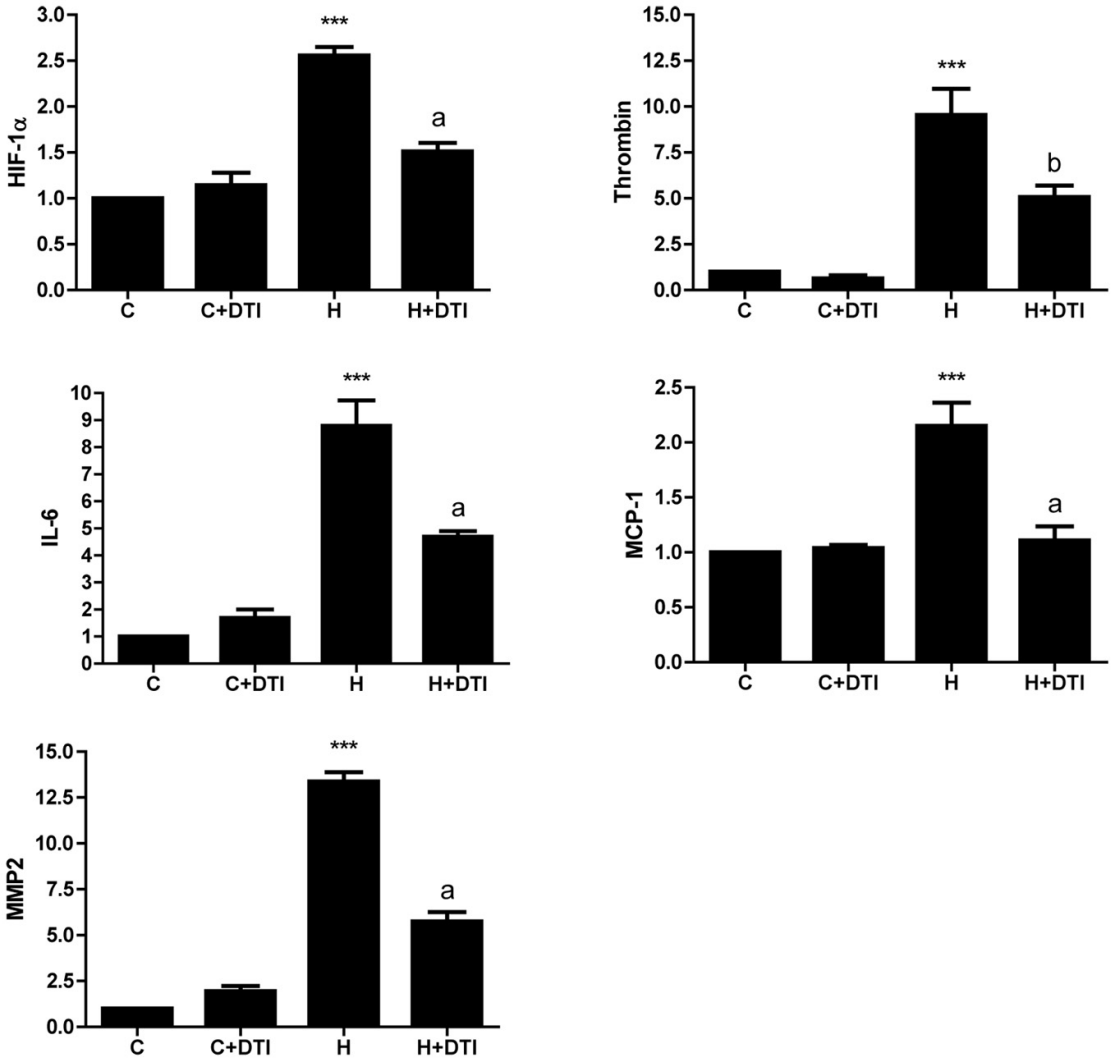
**Brain microvascular endothelial cell cultures were treated with 1 nM direct thrombin inhibitor, dabigatran (DTI) and were exposed to hypoxia for 6 h.** Total RNA was collected, reverse transcribed, and mRNA expression of HIF-1α, thrombin, IL-6, MCP-1, and MMP2 determined by real-time PCR. Data are from four separate experiments performed in triplicate and expressed as fold change over control (C). ^***^p < 0.001 vs. C (control); ^a^*p* < 0.001, ^b^*p* < 0.01 vs. H (hypoxia).

In contrast to the modest effects on cell survival, thrombin inhibition had profound effects on inflammatory gene expression in endothelial cell cultures exposed to hypoxia. Real-time PCR analysis of RNA collected from brain endothelial cells exposed to 6 h hypoxia showed a significant (*p* < 0.001) increase in mRNA for HIF-1α as well as for several inflammatory-associated genes including thrombin, IL-6, MCP-1, and MMP2. Treatment of endothelial cells with the thrombin inhibitor (1 nM) significantly (*p* < 0.01–0.001) reduced hypoxia-mediated effects on inflammatory gene expression (Figure 1).

### Treatment of AD mice with DTI reduces cerebrovascular expression of HIF-1α and inflammatory proteins

**Figure 2:**
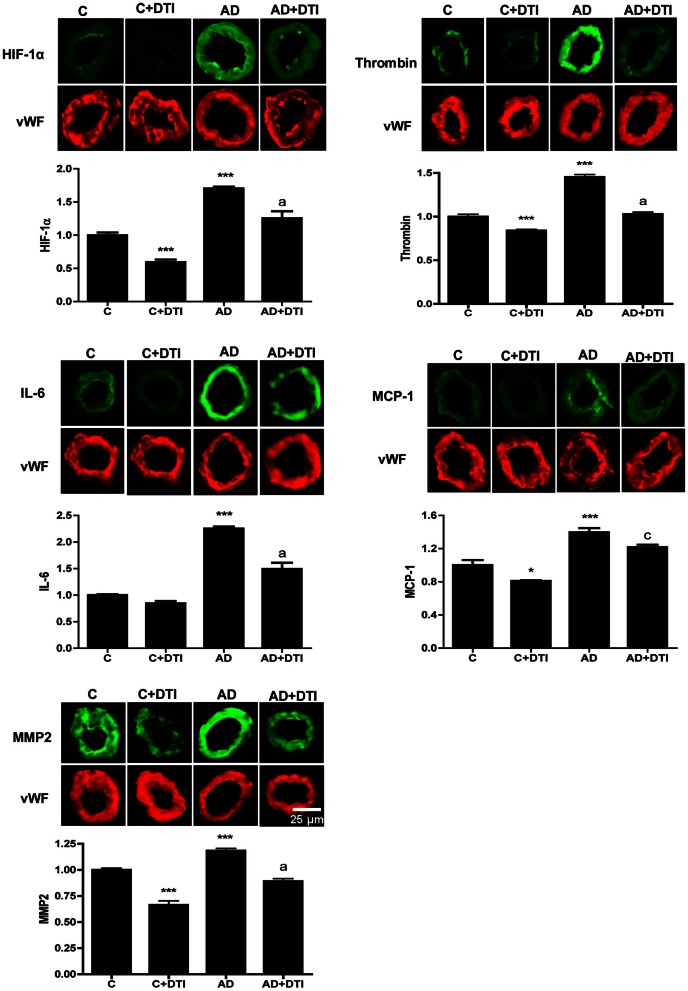
**Brain tissues from control, control + DTI, AD and AD + DTI AD mice were fixed and immunostained with HIF-1α, thrombin, IL-6, MCP-1, and MMP2 primary antibodies and fluorescence labeled secondary antibody (green).** The bar graph denotes signal intensities normalized to endothelial specific marker von Willebrand factor (vWF, red) and control values set to 1. Data are from four mice per group. ^*^p < 0.05, ^***^p < 0.001 vs. C (control); ^a^*p* < 0.001, ^c^*p* < 0.05 vs. AD.

Microvessels from frontal cortex sections were examined by immunofluorescence from control and AD transgenic mice as well as control and AD mice that received 34 weeks of daily DTI administration (100 mg/kg). A comparison between control and AD mice showed that there was a significant (*p* < 0.001) increase in cerebrovascular expression in AD mice of HIF-1α, thrombin, IL-6, MCP-1, and MMP2 (Figure 2). Treatment of AD mice with DTI significantly (*p* < 0.05–0.001) reduced expression of these proteins (Figure 2).

### Dabigatran reduces expression of HIF-1α, thrombin, IL-6, MCP-1, and MMP2 in the brains of AD transgenic mice

**Figure 3:**
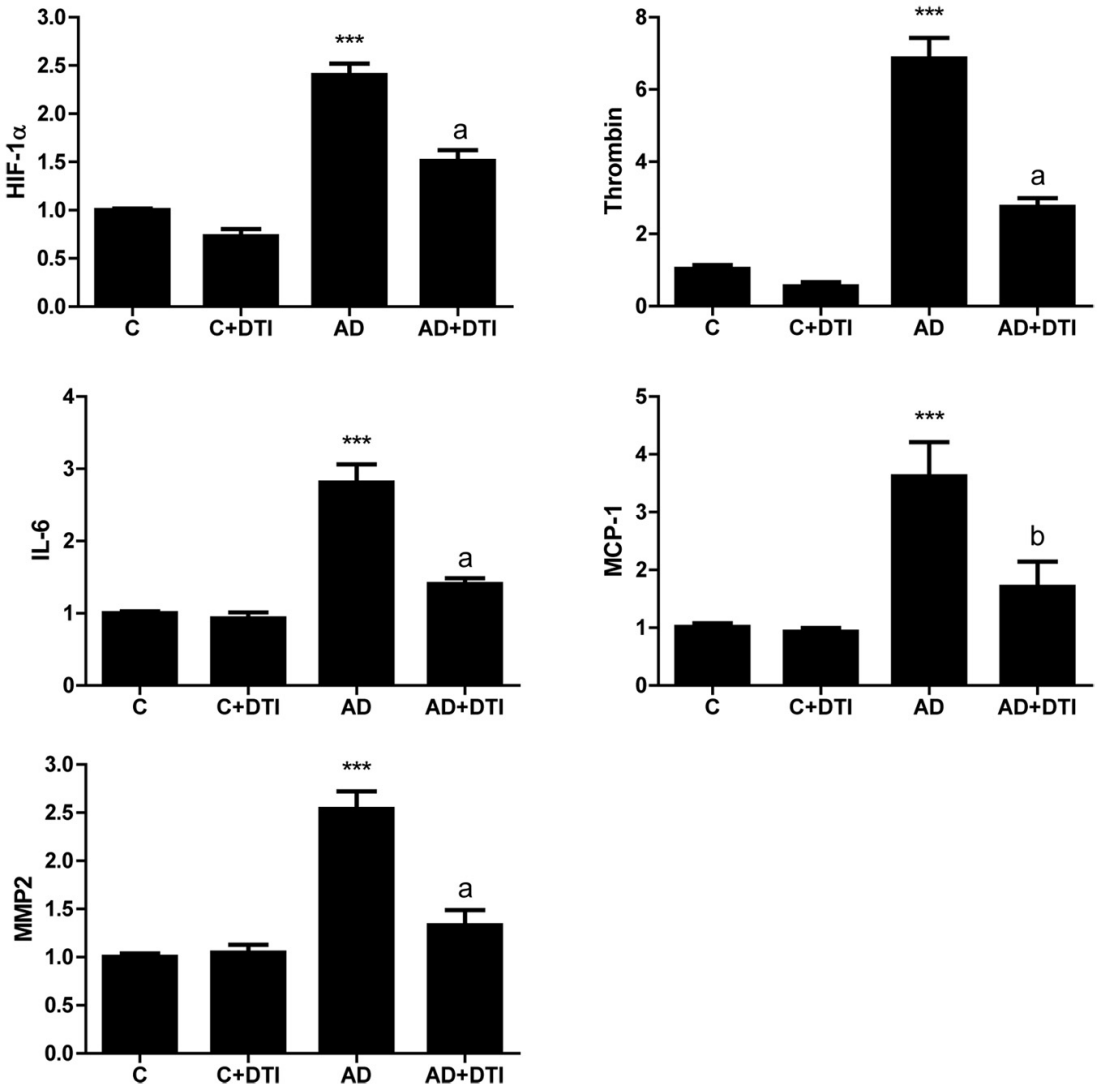
**Brain tissues from control, control + DTI, AD and AD + DTI mice were homogenized, total RNA collected, reverse transcribed and mRNA expression of HIF-1α, thrombin, IL-6, MCP-1, and MMP2 determined by real time PCR.** Data are from four mice per group and expressed as fold change over control (C, untreated). ^***^p < 0.001 vs. C (control); ^a^*p* < 0.001, ^b^*p* < 0.01 vs. AD.

Examination of brain samples from AD transgenic mice by real time PCR demonstrated that expression of RNA for HIF-1α, thrombin, IL-6, MCP-1, and MMP2 was significantly (*p* < 0.001) increased compared to levels in control animals. Similar to the data obtained for cerebrovascular expression (Figure 2), examination of brain tissues also showed a significant (*p* < 0.01 to *p* < 0.001) decrease in RNA levels of these same proteins in AD mice that received DTI (Figure 3).

### Generation of ROS in AD mice and hypoxic endothelial cell cultures is inhibited by dabigatran

**Figure 4:**
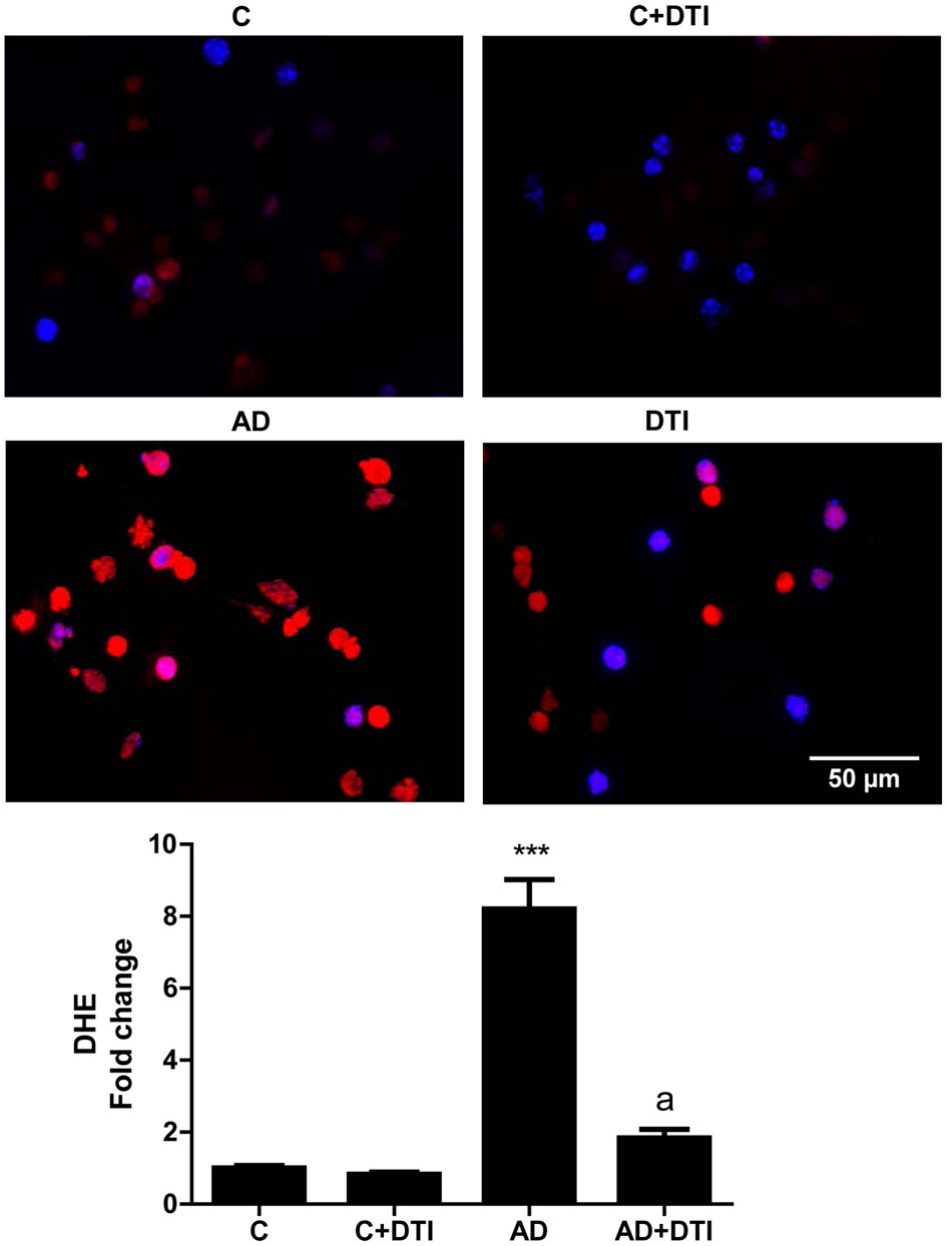
**Brain tissue sections from frontal cortices from control, control + DTI, AD, and AD + DTI mice were incubated for 30 min with 5 μM of Dihydroethidium (DHE, red) fluorescence dye, and NucBlue stain (blue).** Data represents signal intensities of DHE stained cells to non-stained cells. ^***^p < 0.001 vs. control (C); ^a^*p* < 0.001 vs. AD.

The effect of DTI on ROS level in brain tissue sections from frontal cortices of AD transgenic mice was assessed using the fluorescence dye DHE. Quantitation of DHE levels showed that ROS generation was 8-fold higher (*p* < 0.001) in sections from AD mice brains compared to sections from control mice (Figure 4). Administration of DTI for 34 weeks to AD mice significantly (*p* < 0.001) decreased DHE level compared to untreated AD mice (Figure 4).

An examination of cultured brain endothelial cells exposed to 6 h hypoxia showed a significant (*p* < 0.001) increase in ROS levels, as assessed by an increase in DHE fluorescence. Treatment of endothelial cell cultures with DTI blocked (*p* < 0.001) this increase in hypoxia-induced ROS generation (Table 1).

**Table 1:** **ROS levels in cultured brain endothelial cells**.

**Control**	**Control + DTI**	**Hypoxia**	**Hypoxia + DTI**
1.00 ± 0.08	0.65 ± 0.07^***^	1.51 ± 0.19^***^	0.92 ± 0.06^a^

Brain endothelial cell cultures were incubated in serum-free media or serum free media plus DTI (1 nM) and exposed to hypoxia (1% O_2_) or normoxic (21% O_2_) conditions for 6 h. The cells were incubated for 30 min with 5 μM of Dihydroethidium (DHE, red) fluorescent dye and NucBlue stain (blue). ROS generation was assessed by quantitation of signal intensities of DHE stained cells to non-stained cells. Data represent mean signal intensity of DHE stained cells to non-stained cells ± SD from at least 3 separate experiments performed in duplicate.

*** p < 0.001 vs. Control;

a p < 0.001 vs. Hypoxia.

## Discussion

The results of the current study define thrombin as a key mediator in the cerebrovascular response to hypoxia. The data demonstrate that inhibition of thrombin in culture blocks the increase in inflammatory protein expression and ROS generation evoked by hypoxia. Also, administration of the thrombin inhibitor DTI to transgenic AD mice diminishes expression of inflammatory proteins and ROS in the cerebromicrovasculature of those mice. Taken together, these results suggest that inhibiting thrombin generation could have therapeutic value in AD and other disorders where hypoxia, inflammation, and oxidative stress are involved.

The role of vascular risk factors in the development of AD is increasingly recognized. Although risk factors such as hypercholesterolemia, hyperhomocysteinemia, and diabetes may affect the brain in multiple ways, the injurious effects of these risk factors on the cerebrovasculature likely contribute to deleterious events in the AD brain (Knopman and Roberts, 2010; Grammas, 2011). Vascular inflammation is a common mechanistic pathway activated by hypercholesterolemia, hyperhomocysteinemia, diabetes as well as other vascular risk factors (Rojo et al., 2008; Rosenbaum et al., 2012). Hypoxia, thought to trigger degenerative changes in the brain that contribute to the development of dementia, also has profound effects on the inflammatory response of the vasculature (Grammas et al., 2011; Daulatzai, 2012). The cerebrovasculature may be a locus where multiple pathogenic processes converge and contribute to compromised neurovascular function.

Hypoxia and the multifunctional protein thrombin are linked. Hypoxia stimulates angiogenesis and thrombin is a key mediator of the angiogenic process (Dupuy et al., 2003; Green and Karpatkin, 2010; Zhou et al., 2012). Beyond its central role in the coagulation cascade, thrombin can directly affect cellular processes in a variety of cell types including endothelial cells, glia, and neurons (Lee da et al., 2006; Huang et al., 2008; Zundorf and Reiser, 2011; Alabanza and Bynoe, 2012). There is an extensive literature identifying the ability of hypoxia-stimulated peripheral endothelial cells to express a proangiogenic/inflammatory phenotype that is mediated by thrombin (Chen et al., 2010; Sabit et al., 2010). However, the response of peripheral endothelial cells to thrombin may not necessarily reflect the response of the highly specialized endothelial cells that comprise the blood-brain barrier. Using a human cell line, thrombin has been shown to induce an inflammation in brain endothelia (Alabanza and Bynoe, 2012). Also, in rodent-derived brain endothelial cells thrombin can elicit a pro-inflammatory phenotype (Zhou et al., 2012). Studies have shown that brain endothelial cells can both synthesize thrombin as well as respond to exogenously applied protein (Yin et al., 2010). Thrombin could, therefore, function as an autocrine factor that can regulate endothelial cell activation in the brain. The data herein which document the ability of DTI to abrogate the pro-inflammatory effects of hypoxia support a central role for thrombin as a regulator of the cerebrovascular response to hypoxia and contribute to a growing literature that implicates thrombin in neuroinflammatory conditions in the brain.

The ability of hypoxia to induce vascular inflammation has important implications for the pathogenesis of neurodegenerative diseases such as AD which are associated with hypoperfusion and hypoxia. Neuroinflammation has been extensively demonstrated in the AD brain (Niranjan, 2013). Cytokines and other inflammatory proteins are elevated in human AD as well as in transgenic animal models (Rubio-Perez and Morillas-Ruiz, 2012). Retrospective epidemiological studies suggest that a wide variety of non-steroidal anti-inflammatory drugs significantly reduce one's lifetime risk of developing AD (In't Veid et al., 2002; Etminan et al., 2003; Hoozemans et al., 2003) further supporting the importance of neuroinflammation in disease pathogenesis. In AD there is a robust elevation in inflammatory mediators in the cerebral microcirculation. Compared to microvessels from age-matched controls, AD brain microvessels release significantly higher levels of a number of inflammatory factors including nitric oxide (NO), thrombin, TNF-α, transforming growth factor-β (TGF-β), IL, IL-1β, IL-6, IL-8, and MMPs (Grammas et al., 2006, 2011; Thirumangalakudi et al., 2006). Thus, previous work and the data presented in the current study which documents that hypoxia evokes increased cerebrovascular expression of the same inflammatory proteins documented both in human AD and transgenic AD mice strengthens the argument suggesting a role for hypoxia-mediated effects in the pathogenesis of AD.

Release of vascular-derived inflammatory proteins could stimulate/activate neighboring glial cells, both microglia and astrocytes, to release inflammatory proteins as well as noxious ROS and proteases. This noxious neurotoxic cycle could be augmented by vascular-derived thrombin. In addition to thrombin's inflammatory effects on brain endothelia, thrombin could contribute to deleterious and self-perpetuating neuroinflammation via induction of proinflammatory cytokines including IL-1β, IL-6, TNFα in microglia, and astrocytes (Choi et al., 2003, 2008; Lee da et al., 2006; Huang et al., 2008). Our data showing administration of the thrombin inhibitor DTI to AD mice reduces vascular expression of inflammatory proteins further supports an important role for thrombin as a mediator of neuroinflammation.

The results of the current study show that administration of DTI to AD mice reduces ROS generation in the brain, as assessed by an increase in DHE fluorescence. Also, treatment of endothelial cell cultures with DTI blocks hypoxia-induced ROS generation. The generation of ROS by brain endothelial cells in response to hypoxia is, in addition to inflammation, a destructive mechanism that likely contributes to pathologic changes in the AD brain. In this regard, there is a large literature documenting increased oxidative stress and ROS generation in the AD brain (Mecocci et al., 1994; Good et al., 1996; Nunomura et al., 2006). Indeed, elevated oxidative stress is an invariant finding in AD. Markers of oxidative stress such as protein, lipid, and DNA oxidation appear to develop early in the pathogenesis of AD (Feng and Wang, 2012). ROS generation by nearby vascular or glia cells likely exacerbates the injurious effects of neurotoxins such as amyloid beta which can directly evoke oxidative stress in neurons.

The cerebral microvasculature is source of ROS including NO in neurodegenerative diseases such as AD (Dorheim et al., 1994; de la Torre and Stefano, 2000). Numerous studies have suggested a link between oxidative stress and vascular inflammation (Madamanchi et al., 2005). We have previously documented that expression of inflammatory mediators by brain endothelial cells is increased in response to oxidative stress. In that study oxidative stress results in increased vascular expression of IL-6 and IL-8 in apolipoprotein E-deficient mice, demonstrating further the mechanistic links between oxidative stress and vascular inflammation (Evola et al., 2010).

A large body of data suggests that communication between oxidative and inflammatory processes drive a deleterious “feed-forward” cycle that results in injury and cell death in the brain (Yao et al., 2004; Candore et al., 2010). A key mediator of this process could be thrombin. Oxidative stress has been shown to increase thrombin expression in neurons and endothelial cells (Sanchez et al., 2013). Here, we document that the oxidative stress response to hypoxia is regulated by thrombin and that cerebrovascular ROS generation in AD mice is also regulated by thrombin. Thus, thrombin, in addition to its well characterized role as mediator of inflammation, is likely a key protein in the development of oxidative stress under hypoxic conditions and in AD.

The literature suggests a multifaceted role for thrombin in AD pathogenesis. Thrombin *in vitro* can stimulate production of the amyloid precursor protein (APP) and cleavage of APP into fragments that are found in amyloid plaques of AD brains (Igarashi et al., 1992; Ciallela et al., 1999). Thrombin is also important for the proteolytic processing of the microtubule-associate protein tau, a primary component of the neurofibrillary tangle (Arai et al., 2005). Immunoreactivity for the major brain thrombin inhibitor, protease nexin-1 is found to be significantly decreased in AD brains, particularly around blood vessels, highlighting the importance of vascular-derived thrombin (Vaughan et al., 1994). Intracerebral administration of thrombin to rodents increases apolipoprotein E levels and results in neuronal injury and cognitive deficits (Mhatre et al., 2004, 2006).

These data, taken together with the results of the current study showing a key role for thrombin as mediator of hypoxia-induced inflammation and oxidative stress, argue that thrombin inhibition could be a useful strategy for mitigating pathology in AD. In this regard, a study where AD patients receiving donepezil are compared to those receiving donepezil plus the thrombin inhibitor hirudin shows that patients who received hirudin demonstrate improvements in activities of daily living, behavioral and psychological symptoms of dementia as well as cognition compared to patients on donepezil alone (Li et al., 2012). There are many caveats regarding the therapeutic use of thrombin inhibitors for neurodegeneration, such as the potential to cause bleeding. However, the results of the current study showing a key role for thrombin as a central mediator of hypoxic effects on inflammation and oxidative in the cerebrovasculature, argue that exploration to further define the mode of action of thrombin and thrombin inhibitors in diseases such as AD where hypoxia is an important contributing factor to disease pathogenesis is warranted.

### Conflict of interest statement

The authors declare that the research was conducted in the absence of any commercial or financial relationships that could be construed as a potential conflict of interest.
